# Deep Brain Stimulation for Substance Use Disorders? An Exploratory Qualitative Study of Perspectives of People Currently in Treatment

**DOI:** 10.1097/ADM.0000000000001150

**Published:** 2023-03-03

**Authors:** Erika Versalovic, Eran Klein, Sara Goering, Quyen Ngo, Kate Gliske, Marion Boulicault, Laura Specker Sullivan, Mark J. Thomas, Alik S. Widge

**Affiliations:** From the Department of Philosophy, University of Washington, Seattle, WA (EV, EK, SG); Center for Neurotechnology, University of Washington, Seattle, WA (EV, EK, SG); Department of Neurology, Oregon Health and Science University School of Medicine, Portland, OR (EK); Butler Center for Research, Hazelden Betty Ford Foundation, Center City, MN (QN, KG); College of Computing, Massachusetts Institute of Technology, Cambridge, MA (MB); Department of Philosophy, Fordham University, New York, NY (LSS); Department of Neuroscience, University of Minnesota, Minneapolis, MN (MJT); Medical Discovery Team on Addiction, University of Minnesota, Minneapolis, MN (MJT, ASW); and Department of Psychiatry and Behavioral Sciences, University of Minnesota, Minneapolis, MN (ASW).

**Keywords:** deep brain stimulation, substance use disorders, qualitative, neural technology, neuroethics

## Abstract

Although previous studies have discussed the promise of deep brain stimulation (DBS) as a possible treatment for substance use disorders (SUDs) and collected researcher perspectives on possible ethical issues surrounding it, none have consulted people with SUDs themselves. We addressed this gap by interviewing people with SUDs.

**Methods:**

Participants viewed a short video introducing DBS, followed by a 1.5-hour semistructured interview on their experiences with SUDs and their perspective on DBS as a possible treatment option. Interviews were analyzed by multiple coders who iteratively identified salient themes.

**Results:**

We interviewed 20 people in 12-step–based, inpatient treatment programs (10 [50%] White/Caucasian, 7 Black/African American [35%], 2 Asian [10%], 1 Hispanic/Latino [5%], and 1 [5%] Alaska Native/American Indian; 9 women [45%], 11 men [55%]). Interviewees described a variety of barriers they currently faced through the course of their disease that mirrored barriers often associated with DBS (stigma, invasiveness, maintenance burdens, privacy risks) and thus made them more open to the possibility of DBS as a future treatment option.

**Conclusions:**

Individuals with SUDs gave relatively less weight to surgical risks and clinical burdens associated with DBS than previous surveys of provider attitudes anticipated. These differences derived largely from their experiences living with an often-fatal disease and encountering limitations of current treatment options. These findings support the study of DBS as a treatment option for SUDs, with extensive input from people with SUDs and advocates.

Annual drug overdose deaths in the United States rose above 100,000 in 2021, a 28.5% increase from the previous year.^1^ A 2020 national survey estimated that 38.7 million people (14.5%) had a substance use disorder (SUD), with 28.3 million of them alcohol related.^2^ Both nonfatal and fatal overdoses increased during the COVID-19 pandemic.^3^ Amidst this need, current treatment options remain limited.^2,4^ Although inpatient treatment helps people with SUDs achieve initial abstinence, the rate of relapse is high.^5^ Agonist therapies are primarily indicated for opioid use disorders and are often difficult to access. In this context, deep brain stimulation (DBS) is being explored as a potential treatment.

DBS is a surgically invasive therapy commonly used to treat movement disorders such as Parkinson disease and essential tremor, and, more recently, obsessive-compulsive disorder. Electrodes are implanted in the brain and connected to a pulse generator implanted under the chest wall. After recovery from surgery, stimulation is started and then gradually titrated to achieve functional levels over multiple clinic visits. DBS is broadly thought to disrupt brain networks whose overactivity or overconnection gives rise to symptoms.^6^ Because of its initial successes, DBS is being explored as a treatment option for depression, posttraumatic stress disorder, and SUDs.^7,8^

Case reports of experimental DBS for refractory SUDs through stimulating the nucleus accumbens describe notable improvements: alleviation of cravings, reduction of self-reported depression and anxiety, and cessation or significant reduction of use.^9–11^ Clinical trials are exploring the use of DBS for opioid use disorders (eg, NCT03950492, NCT04354077).^12^ Still, enthusiasm has been tempered by concerns from researchers and bioethicists regarding potential coercion in the study DBS for SUDs.^13^ SUDs are socially stigmatized and often criminalized, increasing the risk of pressure to pursue treatments to avoid prosecution. It is also unclear whether individuals would accept a physically invasive neurosurgical intervention for SUDs, given the view that SUDs are fundamentally less disabling or dangerous than other DBS indications.^14,15^

Many limitations on SUD treatment success reflect social stigma and underinvestment, and no technology can substitute for the policy solutions needed to reverse structural inequalities. Nonetheless, even when people can access treatment, recovery is often evasive. For them, DBS may be more attractive than researchers imagine. Indeed, research target population preferences may differ significantly from researcher or clinician perspectives.^16^ Consultation with potential users can reveal important considerations about neurotechnology development to guide research.^17,18^ Based on our prior work, we hypothesized that people with SUDs would view potential risks, benefits, and ethical challenges of DBS differently from clinicians.

Here, we report a qualitative study on the values, interests, and concerns of people with SUDs in relation to the prospect of DBS as a treatment option. We interviewed people in treatment centers in the early abstinence phase of their SUDs, asking about participants' experiences with their disorder and treatment, and their perspectives on DBS across 5 themes: personal agency, social dynamics, stigma, privacy, and interactions with the health care system. These aspects reveal how common concerns surrounding DBS (eg, physical invasiveness and device maintenance) compare with the burdens already experienced through the course of SUDs and their treatment.

## METHODS

We conducted 20 semistructured interviews with people in residential treatment for SUDs. Interview guides were developed collaboratively through a series of discussions among authors, building on previous work related to neurotechnologies^19,20^ and with attention to the particular circumstances of treatment for SUDs. Purposive sampling methods were used for representation of a range of substances and racial groups often excluded from medical device research.^21^ Participants were recruited through the (anonymized for review) treatment system. The study was reviewed by the University of Washington Institutional Review Board (STUDY00009975) and Hazelden Betty Ford Foundation's Internal Research Collaboration Board. Procedures were followed in accordance with our institutional review boards.^22^

Participants were initially asked about their experiences with addiction and treatment. They then watched a 5-minute video introducing DBS (Supplemental Digital Content, Supplementary Video 1, http://links.lww.com/JAM/A415). The interview guide was structured around ethical and social concerns related to the application of DBS to mental health disorders, including agency (eg, how could you imagine a DBS enhancing or undermining a user's sense of agency?), social relationships (eg, would you involve loved ones in the process of getting a DBS?), stigma (eg, how could possible stigma of a neural device interact with stigma surrounding SUDs?), privacy (eg, who should have access to neural data?), and interactions with the health care system (eg, what kind of support is needed for follow-up appointments?). At the end of the interview, participants were asked if DBS would be something they might consider if it became available, who the most appropriate target users (if any) would be, and about the value of gathering target-user perspectives.

Interviews were conducted and recorded through HIPAA-compliant Zoom by EK and EV and lasted 1.5 hours on average. Demographic surveys were administered online postinterview. Participants were compensated $25 through a gift card. Interviews were transcribed using an online service. SG, EK, and EV read the interviews and conducted thematic content analysis. The first 12 transcripts were each independently, inductively coded on atlas.ti, followed by discussions to reconcile code differences to arrive at the final coding scheme. The final 8 were coded by EV. To ensure sensitivity to the specifics of SUD experience and treatment, we used a team-based approach^23^ with monthly meetings of the full authorial team, including our SUDs experts, to check in, discuss any difficulties, and make decisions about the research process (eg, timeline, determining coding schemes, broadening recruitment). Data were collected from September 2020 to May 2021 and analyzed from May 2021 to December 2021. Methods reported here are in line with the consolidated criteria for reporting qualitative research^24^ and qualitative research review guidelines^25^ checklists.

## RESULTS

### Demographics

Participant demographics and specifics regarding primary substance, prior treatment, co-occurring disorders, and family history are presented in Table 1. There was nearly equal representation of female and male participants (11:9, respectively). Ten of the 20 participants were White, 7 identified as Black/African American, 2 as Asian, 1 as American Indian/Alaska Native, and 1 as Hispanic/Latino. The age spread was 25 to 64 years, with education levels from some high school to completion of a graduate degree. The most prominent substance was alcohol (90%), with just under a third identifying marijuana (30%) and 2 to 3 participants identifying each of the categories of opioids, cocaine, and methamphetamines. Seventeen of the 20 participants had a family history of SUDs.

**TABLE 1:** Participant Demographics

	n (%)
Sex	
Male	11 (55)
Female	9 (45)
Nonbinary	0 (0)
Race/ethnicity*	
White or Caucasian	10 (50)
Hispanic or Latino	1 (5)
Black or African American	7 (35)
Asian	2 (10)
American Indian/Alaska Native	1 (5)
Age	
25–34 y	7 (35)
35–44 y	8 (40)
45–54 y	2 (10)
55–64 y	3 (15)
Over 65 y	0 (0)
Education level	
Some high school	3 (15)
Some college	6 (30)
2-y College degree	3 (15)
4-y College degree	6 (30)
Graduate-level degree	2 (10)
Substance*	
Alcohol	18 (90)
Cannabis	6 (30)
Opioids	3 (15)
Cocaine	3 (15)
Methamphetamine	3 (15)
Prescription pills	2 (10)
Co-occurring disorders*	
Depression	7 (35)
Anxiety	5 (25)
Obsessive-compulsive disorder	2 (10)
Other (attention deficit disorder, diabetes, panic disorder)	3 (15)
None disclosed	11 (55)
Prior treatment	
None prior	10 (50)
Intensive outpatient prior	4 (20)
Inpatient once prior	4 (20)
Inpatient more than once	2 (10)
Family history	
Yes	17 (85)
No	3 (15)
Age of first use	
<10 y	1 (5)
10–14 y	8 (40)
15–19 y	8 (40)
>19 y	3 (15)

*Respondents could select more than 1 option; totals may add up to over the number of participants.

### Findings

#### Initial Reaction to DBS: Unfamiliar and Apprehensive, Yet Interested

Most participants initially expressed unease regarding the physically invasive nature of DBS (ie, requiring surgery). First impressions often described DBS as “weird” and “scary”: “wow, it's crazy, because going deep inside the brain is something that you can't really play with” (H12) and “Unlike taking an oral pill or taking a shot, it's invasive. To be honest, that's a little scary” (H14). Despite that initial unease, when prompted at the conclusion of the interview as to whether DBS was something they would ever personally consider, only 1 participant said no outright: “it reminds me of shock therapy … Oh God. I don't want something in my head” (H6).

The majority of participants expressed interest but differed in their perceptions of when DBS would be a reasonable option. Many described seeing DBS as a “last resort,” but there was high variation in where people identified that threshold. Some described being open only if they had exhausted all other existing treatment options, whereas others said that they could see themselves reaching last resort desperation with a single relapse: “if I relapse one more time then yeah, I'm all for it” (H16). Others saw themselves as early adopters: “I would definitely raise my hand to say, ‘Hey, let me jump on ship’” (H12).

#### Perspectives on Living With SUD, Treatment, and DBS

Participants described their experiences living with SUDs and in treatment, and how these experiences shaped their perspectives on the prospect of DBS.

#### Living With an SUD

A common theme was that addiction is difficult to overcome and, all too often, fatal (Table 2). Participants felt a loss of control and at the mercy of their cravings. One participant likened their disorder to a “puppet master” making them do things they could not stop. Participants reported family histories of substance use that involved recurrent relapse, family trauma, and death.

**TABLE 2:** Perspectives on Living With a SUD

SUDs	
Personal agency	“**It's like a puppet master**. Like, “I know I'm moving. I know I'm doing this. Why can't I stop?” because **something else is controlling me**.” (H16) “**I don't make the right decisions when I'm drunk**. So if I'm drunk all the time, ... **I'm just wasting money** on things that I'm not supposed to be wasting on… Maybe I wouldn't have dropped out of school because I started drinking and **I dropped out of school**.” (H20)
Social dynamics	“I didn't know how to make friends before I started using so that's why I sought out drugs… Other than my family, **I haven't really had friendships that didn't involve drugs.**” (H3) “My entire step side of the family, **I can feel the judgment when I walk into the doors**. I mean, they all knew I was a drug addict… People talk about us behind our backs and we know it, and **there's all this judgment that goes on**... I've said many, many things that I regret, things that made me ashamed of who I was, I've broken [their] trust and there's **a lot of things to recover there**… **So I've seen this addiction affect every loved one in my life**.” (H1) “**I shut my whole family out**. I just stopped talking to them. I was embarrassed and I didn't want to be ridiculed. I felt like I didn't want to explain anything to anybody. I just wanted to drink.” (H18)
Stigma	“Because of the stigmatism with it, **I drank to hide who I actually was, so that I could actually have relationships, because I thought that was the only way I'd be able to have them, if people didn't know who I wa**s.” (H6) “There is such a stigma... I used to work in [a clinical setting]. And when an alcoholic would come in they'd be like, **“Oh, put him in the back.”** They're definitely treated differently.” (H9) “My [parent] for example, **“Why can't you just quit drinking?” He does not understand addiction. He believes it's all willpower, stuff like that.”**” (H19) “I feel like I get stereotyped as that typical drunk [ethnicity] guy, at the store buying alcohol first thing in the morning, getting drunk, sitting out all day, just drinking and lazy [ethnicity] stereotype... That actually bugs me because I typically, at least at a minimum put in about a 70-hour work week, easily... I've worked very hard at what I do, but **I do feel like I get stereotyped as that lazy alcoholic minority.**” (H10) “I live in wine country. There are people drinking at 10 o'clock in the morning, and nobody says anything. So, **once you say you're an alcoholic, the way people treat you is going to change rather than, ‘I'm a wine aficionado.’** And I definitely think living near [city] and stuff, the **stigma around like meth, heroin and stuff is a lot greater than alcohol**. Even though it's the same disease just manifested in a different way.” (H9)
Privacy	“When you're going through addiction, **you're hiding almost everything**.” (H10) “I would say my addiction would keep me more private as in holding, like not saying certain things about myself, or **hiding that I went out** and got drunk last night.” (H9) “I **isolated hardcore**. Like, most of the people in my life had, and maybe some of them still do. They have no idea.” (H6)
Interactions with health care system	“So like I said, **bad experience at detox**, literally went there, came out, drank a whole handle and it just got worse…” (H14) “I do believe there is so many beneficial things that the healthcare system does for us that it's just absolutely ignorant to completely distrust them fully. But also it gets to a point where sometimes **the opioid pandemic is kind of started by doctors. They continue to prescribe people with these opioids that they actually don't need**. I mean, if I get my teeth pulled, give me five Vicodin, and I'm fine. Don't give me 30 of them and then three more refills, which that didn't happen to me, but that's just an example of things that I've seen before.” (H2) “In terms of me with my primary care physician, I've moved around a lot for work, so I've bounced different primary care physicians. I never had an ongoing long-term therapist... **Until I came here, I really haven't dealt with healthcare providers.**” (H4)

*Bolding in tables done for emphasis by authors.

Participants recounted struggling with shame. Their SUDs had harmed loved ones, and they felt judged by family members and work colleagues. Nearly all had experienced stigma (eg, SUD understood as lack of willpower or indicative of moral failure). Most participants struggled to maintain relationships and reported isolating themselves to hide their substance use and avoid stigma. They shared struggles with self-trust and guilt about manipulating others when the “addict” part of themselves was operative. In addition to the social costs of SUDs, participants described financial and emotional costs, often with negative consequences for personal and work relationships, financial resources, and self-esteem.

#### Perspectives on SUD Treatment

Participants' sentiments about their treatment and prospect of recovery were often marked by uncertainty, desperation, and determination as they reflected on how much they had lost to their SUD (Table 3). Some were skeptical of their own ability to recover, given that they had watched peers struggle and relapse. A common concern was the unpredictability of cravings: “Honestly, I feel like, once I complete treatment, if I were to have a bad enough day, I could potentially say just screw it and go get a drink and snowball back to where I was or even worse” (H19). Nearly all participants viewed SUDs as long-term diseases and recognized their recovery as fragile. As one participant put it, “12 steps is lifelong. It's forever” (H20). Participants often shared lessons from their 12-step–based programs: the need to rely on others, recognition that they could not maintain abstinence alone, and that, even with support, they still have to “do the work” to maintain abstinence. Many also mentioned the importance of surrendering to a higher power.

**TABLE 3:** Perspectives on SUD Treatment

Treatment	
Personal agency	“**There's some dudes up there, they've been in rehab eight times**. You Can See that they really want to try. It's just life hits you hard, bro. Things happen. You get triggered just like you get... This disease is no joke.” (H20) “​​They always tell you, ‘Depend on your higher power.’ Depend on that power that's higher than yourself, because you can't depend on just you. Because if you could depend on just you to get up out of this, you wouldn't be in this situation of addiction. You wouldn't have the relapses. You wouldn't have the cravings or the urges, if you could just depend on yourself. So **apparently we can't just depend on ourselves or rely on ourselves**. We need that help.” (H12) “A lot of people that have alcoholism, they probably drink most of their money away. And if they're working to stay on their feet, them getting a job, they better put their sobriety first… but bills don't give a damn about that at the end of the day. You know what I'm saying? Bills don't give a f*** about sobriety.” (H20)
Social dynamics	“So I'm **looking forward to mending those relationships sober**. I'm looking forward to that. It's not going to be easy cause I did a lot of wrong stuff and I feel really guilty for a lot of the stuff that I did and I'm going to have to make amends for it and apologize and put myself out there. That's the **hardest part of recovery**... how much you have to put yourself out there so that you can get better, so that you can recover and not drink.” (H18)
Stigma	“Yeah. I feel like there is. I mean, it's like, **“So-and-so's back in treatment again**,”” (H10) “I think **being an inpatient, there's a lot more stigma** than if I would have just said, “I'm going to have to go to therapy for my drinking.” Therapy now is kind of what everybody does.” (H9)
Privacy	“I know that a **big part of being in recovery is to be honest and open** about our addiction and stuff, but I know **a lot of people that want to be private** about them.” (H7) “​​**My addiction counselor had mentioned, ‘Tell as many people as you can** because the more people that are on board with this, the easier it is because you need your people.’ Because in active use, I isolated so much that I had no one really other than my partner.” (H6) “**Just my privacy things**. I'm super concerned, even, to have any mention of this on my medical record, or anything, just because I work in a large hospital, and I know it's easy to get to other people's medical records, and then give them stuff. I don't want people to think I'm a f***ing opioid addict.” (H10)
Interactions with health care system	“I have to focus on my money. **It's my life**, as well. What's more important is actually getting my life. I mean, money comes and goes. My life is once. It's once in a lifetime. But it's **always a financial aspect**, especially when you have to take care of a family or when you're responsible for X amount within a household.” (H12) “**They give you your antidepressants, you got to come back in six weeks to see if it's working for you and then come back three months later**. I mean, anytime you are doing something or taking something that's supposed to continually help you, but there's still risk of side effects, it's absolutely ignorant to not continue to follow up with your healthcare provider.” (H1) “I mean, in this fast paced American lifestyle that we live in, everybody's working 40 plus hours a week, you got kids, you got sports activities, schooling and all this stuff that, I mean, sometimes I don't go to the dentist just because I don't have time to go to the dentist. It's the **fear of going into the healthcare system**, but also the **inconvenience of trying to fit it into our busy, crazy daily schedules**.” (H1)

Unprompted, 5 participants expressed concerns regarding limited treatment options for SUDs. Some had negative experiences with existing treatments (eg, anticraving medications with adverse effects). Financial costs of treatment and the difficulties of finding time in busy schedules were described as burdensome. Participants expressed openness to a variety of methods to achieve recovery, often using the metaphor of “tools in the toolbox” to describe this multifaceted approach.

Whereas many participants emphasized the importance of understanding and minimizing cravings as a recovery goal, even more participants named building social community and repairing relationships as key recovery aims: “It's the isolation part of it. It's crazy because you hear that the opposite of addiction isn't sobriety. The opposite of addiction is connection. It blows my mind how true that is” (H7). A majority of participants also expressed the desire to gain self-understanding and learn how to better process emotions.

#### Perspectives on DBS

Participants expressed concerns regarding DBS risks such as surgical complications (Table 4). These were often balanced against existing concerns regarding the high risks of relapse and a desire to aggressively avoid that possibility. Many participants saw overcoming cravings as the hardest obstacle to recovery and were drawn to the possibility of DBS to help quiet cravings and understand their patterns: “I am hopeful to get over these cravings eventually to regain control, to find out more about the causes of why I'm like this” (H13). Participants who had co-occurring disorders, some of which might also be treatable with DBS, expressed increased interest in DBS if it could simultaneously help them with their depression or obsessive-compulsive disorder.

**TABLE 4:** Perspectives on DBS

DBS	
Personal agency	“Everybody in the AA community knows that **you can't do it yourself**. And if your higher power, it just happens to be some little magical box that they stuck in your head to help refresh your brain once in a while. I think that would actually be embraced.” (H1) “So **now I might have an extra leg up**. I might have an extra tool in my utility belt, but I still got to go out here and do this kung fu... I'm using it, but what if I have a relapse? What if I have a craving or urge? Oh, guess what? I got the device up in me, so it's going to help me with that craving or that urge. And then, from that, I can go back to my book, and I can go back to my steps, or that I can use another tool, my open line of communication with a sponsor, going to a meeting” (H12) “You have to put everything on the table and I think that's the hardest part… I'm hoping that I will make amends. I'm willing and **I don't see how that device can do that**. **You got to kind of, like, make that happen**.” (H18) “**I would be afraid that I would get addicted to using it**... One of those videos about what addiction is, it showed the mouse who was pressing the button. He got stimulated, and somehow, I can't remember, it totally overtook everything. So, I just pictured me as a mouse and not drinking, but then not necessarily getting better.” (H6) “At this point, I just don't trust my brain to make the right decision at all times. If there's an off chance that I might be able to use it to stimulate it kind of like a drug... **I don't want to end up abusing it**. My goal is to get into recovery and have it last... **I'm just not sure at this point I trust myself quite**.” (H15)
Social relationships	“I would probably wear one of those as a **badge of honor**, one of those deep brain things just like, “**See, I did everything I could.**”” (H9) “If anything, **I'd be happy to show people that I'm willing to do whatever it takes to fix what's going on**.” (H11) “My [partner] doesn't have very much experience with it [addiction], so they just don't understand. I know **they just want the madness to stop**. After I finish my phone call with you, I'm going to talk to them about it, and my guess is they would probably applaud me too. Especially if the side effects were minimal, **I think they would push me to do it** [get DBS].” (H10) “Having something visible like if she goes and sees her [parent] and her [parent] sees that and she knows what it's for, could just keep making her angry. Just a **reminder that my daughter's a fuck up to her**. I don't know. Or there's something wrong with you. But no, there's not. Whatever.” (H7) “**If it can help me, wow, I mean, could it really improve my life, quality of life and my relationships? Can it help me stay out of the depths of self-pity and depression and not finding things in life fun?** I mean, I don't know if it helps with dopamine or anything like that, but can I find things, small things enjoyable or does it always have to be a rush or a high, or something like that?” (H15)
Stigma	“Would I want something in my head right now, if everything was considered safe and everything? I think I would pick that as an option. I think I would. Yeah. As long as it had a skinny thingy. I got plenty of hair to cover, but yeah... **I don't want to look like Herman Munster** when I go outside” (14) “And then committing to a device like that, might even take that stigma... **I almost feel like there is more of a stigma on inpatient treatment than there would be with the external device**... Once you attach a medical thing to something, people are like, ‘Well, that's what my doctor told me to do.’” (10) “There's **so many people nowadays just so against prescriptions and pills**...’ I can see some stigmas like, ‘You need to go to the extent of getting a device?’ Well, I feel like that's where it brings light that yes, it is serious enough that technology needs to be involved… **I feel like there'd be more stigma against the medication, or more negativeness to the medication versus the device**, because obviously the device is a much more serious note.” (19)
Privacy	“**If I relapsed**, it's my choice to figure out what I need to do. **I don't want anybody like the relapse police running over to my house**, “I know you just relapsed,” or somebody calling me on the phone saying, “You just relapsed and you just had a drink and you wasn't supposed to.”” (14) “It's like having a service dog or a service animal and they're like, ‘Well what is this animal for?’ First of all, **you don't have to disclose that information but if you feel comfortable enough with someone, you will**.” (12) “If I could take care of those cravings, I would've preferred not to have to be so open and share so much information about myself... I feel like there'd be, **I'd be able to maintain more privacy with the device.**” (19) **“I wouldn't want someone to know I have a thingy in my head**. Unless it was because they're part of my team to make sure I'm safe, to make sure it's helping me out or whatever… I just always **want to be able to tell my own story**.” (6) “[Law enforcement accessing neural data] That I probably do have an issue with. **I have been manipulated by the justice system several times. I have very little trust in them…** I have several [relatives] doing life sentences... I have seen how they apply pressure to people that have absolutely nothing to do with it… **I've never felt a law enforcement officer was there to help me**… **My trust in law enforcement, the entire system and several officers personally, it's absolutely zero**. I'd have a huge problem with that, actually.” (11) “​​So **it's kind of like a house arrest bracelet**, however you can leave the property and things like that. It just monitors the alcohol intake from your sweat... But I do think that is good because I do need those consequences, otherwise I'm just going to fall right back down the rabbit hole. So **if someone is on probation or some sort of anything involving law enforcement, I do believe they should have access to it [neural data]. However, if it's just me being a free bird, I don't think they should be able to**.” (19) “You don't want that to be used against you in court or something like that. **One of the things people mentioned about George Floyd was his substance abuse and that wasn't even the major factor.** Despite a person having addictions or whatever it may have been the case, it was wrong, the action. So **you can't discriminate for that, but people hear something, then they demonize substances**.” (18) “**People feel more comfortable seeing a paramedic more than they would be a police officer**, because when police show up it's more of an aggressive factor.” (17)
Interactions with health care system	“Unfortunately, most alcoholics that are out there are middle-class and poor. **They're not rich**. They don't have a lot of time. Like me, I make okay money, but I got to put in a lot of hours. So once every two weeks, yeah, I'd agree to it. I'd probably mess around with it, but this is all research, like you said. **How long would the thing take?** How long would it take?” (20) “For me, things like more **logistical things would be of importance**. How long is the study? How often do I have to go see a clinician or a doctor or whatever? Where are they located? Especially, I live in [city], so the **traffic's pretty bad**.” (15) “It's just a thing. Not necessarily good or bad, it's **just part of the treatment**. I don't know. **I already see a therapist regularly**, so… I had to meet with the person that does my psych meds like once a month anyways… I don't think there's any medical treatment that I'm aware of where you don't have to check in with the doctor periodically.” (3) “To me, that's [follow-up clinic visits] **just part of the maintenance of it**. I've only been here a week and they've adjusted my medications three times already… if I personally had it, I could see myself getting irritated without the instant fixing of the cravings. I could see that, but it's not like my [anti-craving medication] NAC, started working immediately.” (19) “I know in the African American communities, some people would be okay with it. A lot of people wouldn't. **It's just bad connotations with medicine**. A lot of people would point to Tuskegee but it's many incidents. Where the distrust... But **it would be beneficial for people**, all colors, all types because some people feel like they're powerless against drugs and certain substances, especially alcohol.” (18) “**Money talks, bullshit walks, and this is dealing with the brains**. So you're going to have to come out of pocket... it's all about figuring out how to compensate them because putting something in somebody's head and if they have to make out once every two weeks... Most of these people are like me and they got to work. So compensate them.” (20)

Some participants noted how DBS did not seem drastically different from anticraving medications and might even be better: “Honestly, the DBS kind of sounds more concrete or reassuring than, because I can have a bad day and just say screw it and not take the pill and then succumb to my craving, whereas I can't just take that out of my head” (H19). Concerns about physical invasiveness came up twice as frequently with participants who were in their first time in treatment. A couple participants expressed an openness to using the device but only temporarily and as a last-ditch effort: “Getting all-natural is definitely the end goal, for sure, but if I need something to kickstart it and all else has failed, I'm not opposed to it” (H11).

Participants viewed the possibility of DBS-related stigma as real but potentially less concerning than stigma related to existing SUD treatment: “I don't know why they would view me any differently with one of the devices or taking pills. I would say the pros for this device would be, there's no bottles in my bathroom” (H5). Others compared the stigma associated with the visibility of the device—scarring and visible wires or battery packs—with how visible their disorder had already been to those around them and noted how such stigma would be likely counterbalanced by potential benefits.

Nearly all the participants spontaneously mentioned the idea of DBS being another tool in their recovery toolbox. Several participants, all of whom had been through treatment before and whose SUD had recurred, were concerned about possible overreliance on DBS. They envisioned DBS working in tandem with other recovery support systems rather than as a singularly curative intervention. One participant recommended “Being completely upfront with people, saying like, ‘This is simply just a tool as is all of these 12 steps.’ This is just my 13th tool that kind of gives me a little bit more help” (H1). Some participants said that they would only consider DBS in the case that their SUD recurred after multiple times in treatment, although others recognized that even 1 more episode could be deadly. Interest in DBS was often expressed provisionally “if it works” and hinged upon studies proving it safe and effective. Even with these factors accounted for, participants shared a wide variety of concerns about how device use might be implemented.

Participants recognized that a recording neural device could be viewed as a kind of surveillance. Some participants jokingly referenced conspiracy theories about implanted chips and trackers, even as they acknowledged the potential value of allowing health care professionals and sometimes family members access to DBS data. Others raised concerns about sharing that information, even with close family members. Distrust in law enforcement led to most participants not wanting officers to ever have access to neural data. Conversely, 1 participant with experience wearing an alcohol monitoring bracelet had a positive experience with the bracelet being a helpful accountability mechanism and thought DBS data might serve a similar role.

Some participants were concerned about the financial and time burdens of anticipated DBS programming appointments. Others, however, noted that opioid agonist treatments already often require regular check-ins and the associated burdens of appointments, monitoring, and time off from work. Similarly, participants who worked with therapists were already used to making space in their schedules for appointments and found the regular contact helpful for personal accountability and health maintenance.

#### The Importance of Stakeholder Perspectives (Table 5)

**TABLE 5:** Reflections on the Importance of Patient Perspectives for Technology Development

Importance of Interviews With People Living With SUDs
“I mean, no offense to any researchers and doctors, I guess, you probably shouldn't take offense, but a lot of you guys aren't drug addicts and alcoholics. **And as much as you can understand about the brain and how these things work, you haven't actually experienced these things**.” (1)
“If you think that this treatment option is going to be successful for addicts or other people with substance abuse, you should engage them ... To get their feedback on how they would view it, how they may, just to make sure it's actually going to work. **Rather than investing in going down this path and then finding that a lot of people would never be open to this type of treatment.**” (4)
“You want to have these conversations. These are the things you really want to do because, **the more involved they are in a process like this, the better the outcome will be**… You want somebody that can actually A, understand you, B, relate to you, C, help you do it. So these conversations are more than helpful.” (12)
“I think involving people who are dealing with addiction or have family members who are dealing with addiction, **looping them in even when this is just a possibility and not even a current treatment would be very helpful** ... With technologies, there's a level of expertise needed to truly understand it. And so, the common man or woman, they either have to have trust from their provider, or they're going to have to build a pretty extensive understanding of it, or some combination of those things to buy in, in my perspective. **Knowing the population and their ability to understand neurological treatment, I think the more trust you can build over time, the better. After talking to you, I would feel more comfortable**.” (12)
“A lot of people do not understand alcoholism or substance abuse. They just don't. And unless you've been in that you won't know. **Even if they say they do from observation, looking on the outside in is not the same as a person that's been going through it physically, mentally, all of that**.” (18)
“**I feel honored to be part of trying to figure this thing out**.” (5)

Reflecting on the interviews themselves, most participants saw them as critical for informing the research process by incorporating perspectives derived from personal experience. Some participants highlighted the importance of collecting a wide range of perspectives, to prevent overgeneralization. Others framed the interviews as serving an important outreach function of helping inform people with SUDs about the prospect of DBS.

## DISCUSSION

People in treatment for SUDs are open to the possibility of DBS, despite initial apprehensions regarding its physical invasiveness and novelty. This openness arose from the difficulties and high burdens participants faced from their SUDs, and their frustrations with access to and effectiveness of existing treatment options. These responses diverged from prior studies of clinicians and researchers that advocate for more restrictive “last resort” criteria.^14,15^ Participants viewed SUDs as serious diseases that need better treatment options. In the context of prior experience, many considered the risks of DBS well balanced against the potential benefits.

Our findings emphasize that, although DBS may be novel, the considerations it raises surrounding agency, stigma, privacy, and accessibility are not. Participants' experiences of feeling at the mercy of their cravings led them to be interested in the possibility of DBS helping quiet those urges. Their experiences with SUD treatment stigma led them to feeling less concerned about the potential stigma of a visually noticeable neural device. Device maintenance appointment burdens were often not viewed as a potential barrier, given participants' familiarity with frequent therapy and medical appointments and the 12-step emphasis on needing to “do the work” that recovery requires. Participants' view of DBS as “another tool in the toolbox” that, although holding promise, could never do the complex and expansive work that recovery often requires suggests the importance of DBS clinical trials providing additional recovery supports.

Previous work on prospective user acceptability proposes that the degree to which an intervention is considered appropriate relies not only on whether it works but also the perceived burden, opportunity costs, and ethicality (alignment with the user's value system).^26^ Our findings affirm the relevance of these considerations and offer additional insight for how models of acceptability should consider these aspects relative to the preexisting treatment landscape (eg, opportunity costs of inpatient treatment, 12-step emphasizing “you can't do it alone”). Even skeptical participants who expressed initial reluctance about DBS noted that their thinking could shift in the event of a relapse, recognizing the potential fatality of such episodes. Their histories of SUD-related loss among their friends and family highlight the precarity of living with an SUD.

These findings show the importance of consulting those who have the targeted disorder to better understand the ethical issues surrounding novel interventions like DBS. Contra Carter et al.,^27^ we found that people in SUD treatment, for alcohol and for opioids, view addiction as “deadly” and available addiction treatments (eg, anticraving medications) as either ineffective or causing undesired side effects. Many participants are living with co-occurring disorders proposed as clinical trial exclusions.^14^ Given that many people in our study and the broader SUD population have co-occurring disorders,^28^ these exclusions should be reconsidered. The potential reduction of confounding variables may not be justified if the data are inapplicable to the majority of people with severe SUDs.^29^ Indeed, people who have treatment-refractory SUD with frequent relapses may be more likely to live with co-occurring disorders.^30^ There is also reason to expect dual benefit; the most studied DBS target for SUDs (the nucleus accumbens and surrounding white matter) also relieves depressive and anxious symptoms.^31,32^

This study highlights 2 additional considerations: (1) the importance of increased sensitivity to family dynamics that may complexify caregiver considerations and (2) the need for increased data protections to prevent further criminalization of SUDs. Participants often named stronger boundary building with loved ones and community rebuilding as goals for recovery. For some, this meant cutting off familial ties and carefully building a new kind of family. Trials of DBS for psychiatric disorders often expect participants to have at least 1 family member involved with their care and to provide support.^33^ Heightened attention to family dynamics in SUD populations will be needed in clinical trial design.

Second, people with SUDs often experience limitations on and threats to privacy because of the degree of criminalization and stigma of SUDs.^34^ Limitations on privacy occur, for instance, through drug monitoring.^35,36^ Although much of the discussion of privacy in the context of novel neurotechnology, like DBS, has focused on data security,^37^ data ownership,^38^ or agency,^39^ our findings suggest that privacy related to DBS and the criminal justice system is an underappreciated concern for SUDs. The majority of participants said that law enforcement should never have access to neural data. Both issues require careful consideration should DBS go to clinical trials.

Our study has 3 main limitations. Participants were drawn from 2 clinics within the same treatment system, and all expressed positive experiences with their current treatment. This could have led to more positive appraisals regarding the potential of DBS and research more broadly. Furthermore, although we extended recruitment to achieve higher racial diversity, we undersampled many minoritized perspectives (eg, Asian, Native American, Latinx, and Queer identities). Finally, discussion surrounding DBS was hypothetical, which might allow for stronger framing effects from the video and interview questions. As such, we echo other calls for future work to address these perspective gaps.^17,40^

Ultimately, our study shows the importance of understanding novel therapies in the context of the specific features of a disorder, how it is experienced by people who are differently socially positioned, what their treatment options are, and how treatment affects their perspectives on themselves. Addressing the challenges of SUDs will require a multipronged approach that makes use of a variety of intervention and support strategies. Participants' openness to DBS as one “tool in the toolbox” for SUD treatment is notable but should be considered against the backdrop of substantially unequal access to existing forms of treatment and support, and pressing social problems that contribute to and exacerbate the experience of SUDs. DBS may be able to help some people make significant strides in their recovery, but it cannot address all the broader challenges those with SUDs face in our current social context.

## Supplementary Material

SUPPLEMENTARY MATERIAL

## References

[1] CDC, National Center for Health Statistics. Drug Overdose Deaths in the U.S. Top 100,000 Annually. *CDC, National Center for Health Statistics*. https://www.cdc.gov/nchs/pressroom/nchs_press_releases/2021/20211117.htm. Accessed November 14, 2022.

[2] Substance Abuse and Mental Health Services Administration. Highlights for the 2020 National Survey on Drug Use and Health. https://www.samhsa.gov/data/sites/default/files/2021-10/2020_NSDUH_Highlights.pdf. Accessed November 14, 2022.

[3] Shreffler J Shoff H Thomas JJ, . Brief report: The impact of COVID-19 on emergency department overdose diagnoses and county overdose deaths. *Am J Addict*. 2021;30(4):330–333. 33738889 10.1111/ajad.13148 PMC8250732

[4] Mojtabai R Mauro C Wall MM, . Medication treatment for opioid use disorders in substance use treatment facilities. *Health Aff (Millwood)*. 2019;38(1):14–23. 30615514 10.1377/hlthaff.2018.05162 PMC6816341

[5] Andersson HW Wenaas M Nordfjærn T. Relapse after inpatient substance use treatment: a prospective cohort study among users of illicit substances. *Addict Behav*. 2019;90:222–228. 30447514 10.1016/j.addbeh.2018.11.008

[6] Sullivan CRP Olsen S Widge AS. Deep brain stimulation for psychiatric disorders: from focal brain targets to cognitive networks. *Neuroimage*. 2021;225:–117515. 10.1016/j.neuroimage.2020.117515 PMC7802517 33137473

[7] Mahoney JJ 3rd Koch-Gallup N Scarisbrick DM, . Deep brain stimulation for psychiatric disorders and behavioral/cognitive-related indications: review of the literature and implications for treatment. *J Neurol Sci*. 2022;437:120253. 35460949 10.1016/j.jns.2022.120253

[8] Chang R Peng J Chen Y, . Deep brain stimulation in drug addiction treatment: research progress and perspective. *Front Psych*. 2022;13:858638. 10.3389/fpsyt.2022.858638 PMC9022905 35463506

[9] Kuhn J Moller M Treppmann JF, . Deep brain stimulation of the nucleus accumbens and its usefulness in severe opioid addiction. *Mol Psychiatry*. 2014;19(2):145–146. 10.1038/mp.2012.196 23337942

[10] Müller UJ Sturm V Voges J, . Nucleus accumbens deep brain stimulation for alcohol addiction — safety and clinical long-term results of a pilot trial. *Pharmacopsychiatry*. 2016;49(4):170–173. 27145161 10.1055/s-0042-104507

[11] Davidson B Giacobbe P George TP, . Deep brain stimulation of the nucleus accumbens in the treatment of severe alcohol use disorder: a phase I pilot trial. *Mol Psychiatry*. 2022;27:3992–4000. 35858989 10.1038/s41380-022-01677-6

[12] Qu L Ge S Li N, . Clinical evaluation of deep brain stimulation of nucleus accumbens/anterior limb of internal capsule for opioid relapse prevention: protocol of a multicentre, prospective and double-blinded study. *BMJ Open*. 2019;9(2):e023516. 10.1136/bmjopen-2018-023516 PMC6398661 30765398

[13] Pisapia JM Halpern CH Muller UJ, . Ethical considerations in deep brain stimulation for the treatment of addiction and overeating associated with obesity. *AJOB Neurosci*. 2013;4(2):35–46. 29152408 10.1080/21507740.2013.770420 PMC5687095

[14] Ali R DiFrancesco MF Ho AL, . Attitudes toward treating addiction with deep brain stimulation. *Brain Stimul*. 2016;9(3):466–468. 27066935 10.1016/j.brs.2016.03.009

[15] Lee KE Bhati MT Halpern CH. A commentary on attitudes towards deep brain stimulation for addiction. *J Neurol Neuromedicine*. 2016;1(8):1–3. 10.29245/2572.942x/2016/8.1093 PMC5469439 28620655

[16] Anderson KD. Targeting recovery: priorities of the spinal cord-injured population. *J Neurotrauma*. 2004;21(10):1371–1383. 15672628 10.1089/neu.2004.21.1371

[17] Goering S Brown TE McCusker D, . Integrating equity work throughout bioethics. *Am J Bioeth*. 2022;22(1):26–27. 10.1080/15265161.2021.2001108 PMC9514226 34962194

[18] Collinger JL Boninger ML Bruns TM, . Functional priorities, assistive technology, and brain-computer interfaces after spinal cord injury. *J Rehabil Res Dev*. 2013;50(2):145–160. 23760996 10.1682/jrrd.2011.11.0213 PMC3684986

[19] Versalovic E Klein E. “Who will I be?”: relational identity, living with amyotrophic lateral sclerosis, and future-oriented decision making. *Camb Q Healthc Ethics*. 2020;29(4):617–629. 32892772 10.1017/S0963180120000365

[20] Goering S Klein E Dougherty DD, . Staying in the loop: relational agency and identity in next-generation DBS for psychiatry. *AJOB Neurosci*. 2017;8(2):59–70.

[21] Fox-Rawlings SR Gottschalk LB Doamekpor LA, . Diversity in medical device clinical trials: do we know what works for which patients? *Milbank Q*. 2018;96(3):499–529. 30203600 10.1111/1468-0009.12344 PMC6131322

[22] World Medical Association. World Medical Association Declaration of Helsinki: ethical principles for medical research involving human subjects. *JAMA*. 2013;310(20):2191–2194. 24141714 10.1001/jama.2013.281053

[23] Giesen L Roeser A. Structuring a team-based approach to coding qualitative data. *Int J Qual Methods*. 2020;19:160940692096870.

[24] Buus N Perron A. The quality of quality criteria: replicating the development of the consolidated criteria for reporting qualitative research (COREQ). *Int J Nurs Stud*. 2020;102:103452–103452. 31726311 10.1016/j.ijnurstu.2019.103452

[25] Neale J West R. Guidance for reporting qualitative manuscripts. *Addiction*. 2015;110(4):549–550. 25771688 10.1111/add.12857

[26] Sekhon M Cartwright M Francis JJ. Acceptability of healthcare interventions: an overview of reviews and development of a theoretical framework. *BMC Health Serv Res*. 2017;17(1):88–88. 28126032 10.1186/s12913-017-2031-8 PMC5267473

[27] Carter A Bell E Racine E, . Ethical issues raised by proposals to treat addiction using deep brain stimulation. *Neuroethics*. 2011;4(2):129–142.

[28] Han B Compton WM Blanco C, . Prevalence, treatment, and unmet treatment needs of US adults with mental health and substance use disorders. *Health Aff (Millwood)*. 2017;36(10):1739–1747. 28971918 10.1377/hlthaff.2017.0584

[29] Compton WM Thomas YF Stinson FS, . Prevalence, correlates, disability, and comorbidity of DSM-IV drug abuse and dependence in the United States. *Arch Gen Psychiatry*. 2007;64(5):566–576. 17485608 10.1001/archpsyc.64.5.566

[30] Najt P Fusar-Poli P Brambilla P. Co-occurring mental and substance abuse disorders: a review on the potential predictors and clinical outcomes. *Psychiatry Res*. 2010;186(2):159–164. 20728943 10.1016/j.psychres.2010.07.042

[31] Sullivan LS Klein E Brown T, . Keeping disability in mind: a case study in implantable brain-computer interface research. *Sci Eng Ethics*. 2018;22:479–504. 10.1007/s11948-017-9928-9 28643185

[32] Widge AS Malone DA Jr. Dougherty DD. Closing the loop on deep brain stimulation for treatment-resistant depression. *Front Neurosci*. 2018;12:175. 29618967 10.3389/fnins.2018.00175 PMC5871707

[33] Widge AS Dougherty DD. Managing patients with psychiatric conditions treated with deep brain stimulation. In: Ostrem JL Marks WJ, eds. *Deep Brain Stimulation Management*. 3rd ed. Cambridge, UK: Cambridge University Press; 2022:198–214.

[34] Association of women's health, obstetric and neonatal nurses. Criminalization of pregnant women with substance use disorders. *J Obstet Gynecol Neonatal Nurs*. 2015;44(1):155–157.

[35] Polles A Bundy C Jacobs W, . Adaptations to substance use disorder monitoring by physician health programs in response to COVID-19. *J Subst Abuse Treat*. 2021;125:–108281. 10.1016/j.jsat.2021.108281 PMC7789824 34016294

[36] Chang G. Maternal substance use: consequences, identification, and interventions. *Alcohol Res*. 2020;40(2):6. 10.35946/arcr.v40.2.06 PMC7304408 32612898

[37] Bonaci T Calo R Chizeck HJ. App stores for the brain: privacy and security in brain-computer interfaces. *IEEE Technol Soc Mag*. 2015;34(2):32–39.

[38] Naufel S Klein E. Brain-computer interface (BCI) researcher perspectives on neural data ownership and privacy. *J Neural Eng*. 2020;17(1):016039–016039. 31766026 10.1088/1741-2552/ab5b7f

[39] Schönau A Dasgupta I Brown T, . Mapping the dimensions of agency. *AJOB Neurosci*. 2021;12:172–186. 33764258 10.1080/21507740.2021.1896599 PMC8434765

[40] Shen FX. Racial injustice and neuroethics: time for action. *AJOB Neurosci*. 2020;11(3):212–216. 32716756 10.1080/21507740.2020.1778133

